# Costs and outcomes in Finnish heart failure patients treated with left ventricular assist device or heart transplant

**DOI:** 10.1093/eschf/xvag063

**Published:** 2026-02-25

**Authors:** Riina Kandolin, Sami Pakarinen, Paavo Koivistoinen, Saara Tiainen, Jan Kiss, Deirdre Blissett, Markku Pentikäinen

**Affiliations:** Helsinki University Hospital, Haartmaninkatu 4, 00290 Helsinki, Finland; University of Helsinki, PO Box 3, 00014 Helsinki, Finland; Helsinki University Hospital, Haartmaninkatu 4, 00290 Helsinki, Finland; University of Helsinki, PO Box 3, 00014 Helsinki, Finland; Helsinki University Hospital, Haartmaninkatu 4, 00290 Helsinki, Finland; University of Helsinki, PO Box 3, 00014 Helsinki, Finland; Helsinki University Hospital, Haartmaninkatu 4, 00290 Helsinki, Finland; University of Helsinki, PO Box 3, 00014 Helsinki, Finland; Helsinki University Hospital, Haartmaninkatu 4, 00290 Helsinki, Finland; University of Helsinki, PO Box 3, 00014 Helsinki, Finland; MedTech Economics, Winchester, UK; Helsinki University Hospital, Haartmaninkatu 4, 00290 Helsinki, Finland; University of Helsinki, PO Box 3, 00014 Helsinki, Finland

**Keywords:** Advanced heart failure, Left ventricular assist devices (LVAD), Heart mate 3, Heart transplant (HTx), Cost analysis, Survival analysis

## Abstract

**Aims:**

This real-world, retrospective study aimed to evaluate clinical outcomes and healthcare costs in advanced heart failure (HF) patients treated at Helsinki University Hospital with heart transplantation (HTx) or elective or urgent left ventricular assist device (LVAD) therapy over 3 years.

**Methods and results:**

Data were extracted from electronic medical records and validated through clinician review. Patients (*n* = 78) were categorized into three groups: Group 1, HTx as first procedure, stratified into those without (1a, *n* = 25) and with (1b, *n* = 11) prior LVAD; Group 2, elective LVAD (*n* = 30); and Group 3, urgent LVAD (*n* = 12). Study endpoints included survival, 6-minute walk test (6MWT) results, and healthcare costs at 3, 6, 12, and 24 months. Outcomes and costs were indirectly compared to explore their implications for future patient selection strategies.

Survival exceeded 80% in groups 1 and 2. Group 1a had a 24-month survival rate of 84.0% (95% CI: 0.628–0.937), with most deaths (3 of 4) occurring within the first 3 months. Group 1b showed 100% survival throughout follow-up, and group 2 stabilized at 93.4% (95% CI: 0.759–0.983) after two early deaths. Group 3 had a progressive decline to 62.5% at 24 months (95% CI: 0.268–0.846). The confidence intervals between these groups overlap due to the small sample size in group 3. Observed 6-minute walking test (6MWT) performance improved steadily over the first year in all groups, with increases in distance walked and percentage of predicted values observed increasing between baseline and 12 months.

Most healthcare expenses were concentrated within the first 3 months post-surgery. At 3 months, median costs per patient were €177 380 [IQR €121 900] (1a), €207 826 [IQR €83 398] (1b), €187 558 [IQR €67 664] (2), and €293 355 [IQR €67 664] (3). Group 3 incurred significantly higher costs compared to groups 1a (*P* = .004) and 2 (*P* = .003). While no significant difference was observed between group 3 and group 1b (*P* = .335), a difference was observed when group 1a and b were pooled. These trends were consistent at 6 months. The differences were no longer statistically significant at 12 and 24 months, which may be due to wider cost variation or diminishing sample size.

**Conclusion:**

Elective LVAD in patients with advanced HF offers survival outcomes comparable to HTx and incurs similar costs and is preferable to urgent LVAD, which is associated with higher costs and may lead to poorer outcomes. These findings support more proactive patient selection and care pathway optimization in advanced HF.

## Introduction

Heart failure (HF) is a leading cause of hospitalizations in Finland, with severe cases linked to poor prognosis and high healthcare costs.^1,2^ Patients with HF stay 3.7 days longer in hospital compared to matched controls with patients admitted for other reasons. Furthermore, this group had substantially higher 5-year mortality rates, with age being the strongest predictor.^3^

The treatment options for advanced HF include heart transplantation (HTx) and left ventricular assist devices (LVADs), in addition to optimal medical therapy.^4^ In carefully selected patients, HTx is demonstrated to reduce morbidity and mortality.^5^ Nonetheless, the scarcity of donor hearts and contraindications for HTx are substantial limitations. Furthermore, long-term survival may be reduced by acute and chronic rejection, and immunosuppressive therapy following HTx is associated with high costs and potential adverse effects, including infections and malignancies.^6^

Implantation of an LVAD also improves survival in select patients with advanced HF^7,8^ and may serve as either a bridge to transplant (BTT) for those on the organ waiting list or destination therapy (DT), a long-term treatment for patients ineligible for HTx. Limitations of LVAD use include the high procedure and device costs and the risk of complications such as hemocompatibility issues, infections, arrhythmias, and right ventricular failure.^9^ While first-generation LVADs were associated with risk of repeat hospitalizations^10,11^ the MOMENTUM 3 multicentre randomized study, which followed patients for up to 5 years, found that the HeartMate 3 (HM3), a newer model of LVAD using a centrifugal-flow pump, requires fewer pump replacements and demonstrated superior survival and reduced risk of complications or reoperation for device malfunction compared to HeartMate 2, a traditional axial-flow LVAD device.^12^ The 5-year follow-up of the MOMENTUM 3 study reported a survival rate of 58.4% with HM3, compared to 43.7% in the axial-flow LVAD devices arm.^13^ Additionally, real-world studies report 5-year survival rates exceeding 60% and demonstrate that significant improvements in functional capacity and quality of life are sustained over this period.^14^ This strategy is particularly beneficial for patients who are inotrope-dependent or ineligible for heart transplantation, allowing LVAD therapy to be a viable option for destination therapy in these cases.^9^

Economic studies comparing the cost-effectiveness of LVADs to HTx, and medical management remain limited worldwide, and much of the economic evidence is based on older LVAD models.^15^ An economic analysis conducted from a United Kingdom (UK) payer perspective, using clinical outcomes extrapolated from the MOMENTUM 3 study, and prior analyses to make an indirect comparison with medical management, found that LVAD therapy with HM3 is cost-effective in transplant-ineligible patients.^16^

The EUneHTA report^17^ investigating common evidence gaps identifies uncertainties in LVAD cost-effectiveness studies. Key concerns raised include device durability, hospital readmissions, multidisciplinary care, and patient selection, all critical for optimizing outcomes and costs. This underscores the need for more real-world studies that report local costs and compare cost-effectiveness with alternative treatment strategies.

Finland is a country of 5.64 million inhabitants with a publicly funded health care system without significant private sector or insurance-based care. Particularly, surgical heart failure treatment is fully reimbursed, and medical therapy is reimbursed after a relatively low annual cut-off. Advanced heart failure care is centralized in five tertiary university hospitals, and all organ transplantations and LVAD implantations are performed in Helsinki University Hospital. These factors create an applicable setting for cost analyses. This real-world analysis of costs and outcomes among advanced HF patients in Finland aims to support future decision-making, optimizing patient outcomes and resource allocation.

## Methodology

### Study design

This single-centre, retrospective cohort study reports real-world costs and outcomes amongst advanced HF patients treated at Helsinki University Hospital with either HTx or LVAD between 1 January 2022, and 1 January 2025, following the clinic’s decision to exclusively use of HM3 for LVAD procedures. The last date of the first procedure was 15 October 2024. Data was extracted in April 2025 and includes costs and survival data up to this point.

Patient data, including demographics, clinical characteristics, clinical outcomes, and healthcare resource utilization and costs, were extracted from hospital electronic records through data lake queries, with clinicians reviewing case notes where there was uncertainty.

### Participants and study groups

The cohort studied was stratified into three groups:

- Group 1 (HTx group): Patients who underwent either elective or urgent HTx as their first procedure during the study period. This group includes individuals with a prior LVAD implanted before 2022 or in another setting.
- Group 2 (Elective LVAD group): Patients who underwent elective LVAD implantation, with or without subsequent HTx during the study period.
- Group 3 (Urgent LVAD group): Patients who underwent urgent LVAD implantation, with or without subsequent HTx during the study period, where urgent status was defined as being on Extracorporeal Membrane Oxygenation (ECMO). Criteria for ECMO, respectively, were cardiogenic shock not responsive to inotropes, vasopressors, IABP, and coronary intervention in cases of acute MI.

Direct comparisons between treatment groups were not appropriate due to inherent differences in prognosis, particularly for patients requiring urgent intervention. Furthermore, eligibility criteria for LVAD and HTx may influence both survival outcomes and healthcare utilization. However, indirect comparisons between treatment pathways among clinically similar patients offer valuable insights to inform decision-making. To facilitate cost and outcome comparisons between elective LVAD and HTx, Group 1 was further stratified into two subgroups: patients who underwent HTx as a first procedure and those who had received an LVAD before the study period. The sample size in all groups decreases over time as patients were enrolled to the study at different time-points over the 3-year period.

### Survival

Survival was defined as the time from the index procedure to death, and patients were censored at their last known follow-up date if death had not occurred by the end of the study period. Survival analyses were conducted using the Kaplan–Meier method, implemented in Python, to generate survival curves for each treatment group. Uncertainty around these estimates was quantified by calculating 95% confidence intervals over time. Comparisons between groups were assessed visually and supported by differences in survival probabilities at 12 and 24 months.

### 6-minute walk test (6MWT)

Submaximal exercise capacity was assessed using the 6-minute walk test (6MWT) to evaluate functional capacity and overall cardiovascular health.^19^ Urgent patients were unable to perform the test before the index procedure. In elective patients, this test was performed within 3 months before. Change in 6MWT was evaluated using descriptive statistics and reported at 3 months (included tests between 2 and 4 months) and at 12 months (included tests between 9 and 15 months) after the index procedure. Patient follow-up during this period was variable, which influenced the timing and availability of test data.

### Healthcare utilization and expenditure analysis

Resource use and costs were analysed from 3 months before HTx or LVAD implantation to consider treatment expenses associated with preparing patients for implantation and treating advanced HF. For example, in critically ill patients, ECMO may serve as a bridge HTx or LVAD, allowing time for recovery of cardiac function before implantation.^18^ These data were only available for patients treated at Helsinki University Hospital before implantation. The costs for the remaining patients were recorded from the point of admission for LVAD or HTx, and sample sizes for each time-point are reported. Due to this variation, these prior costs are not included in the cumulative mean and median costs.

Hospital costs were categorized using over 31 cost codes and grouped into 6 categories. Mean costs are reported at each time-point, broken down by category. Sample size is recorded for each period, where patients were included if they were alive and had follow-up costs collected in each 3-month time period.

Individual patient cost curves were visually inspected, and the normality of cost distributions at each time point was assessed using the Shapiro-Wilk test. The data were found to be skewed at all time points. Accordingly, median costs at 3, 6, 12, and 24 months were calculated, and the non-parametric Kruskal–Wallis tests were applied to evaluate differences across all groups, followed by *post hoc* testing for pairwise comparisons. Median costs are expected to better reflect the typical cost trajectory associated with HF patients, where individual patients may experience one-time, high-cost spikes, for example, where LVAD patients later have HTx. Costs beyond 24 months post-index procedure were not reported, as the research team determined that the sample size was insufficient for meaningful analysis.

Two sensitivity analyses were performed, repeating the statistical tests. The first pooled group 1a and group 1b, and the second included costs from the 3 months before the index procedure in the calculation of median costs at each time point. Differences between in-hospital treatment times in urgent vs elective LVAD cases was calculated with Mann–Whitney *U* test, and RVAD/dialysis need with Fisher`s exact test.

## Results

### Patient flow

**Figure 1:**
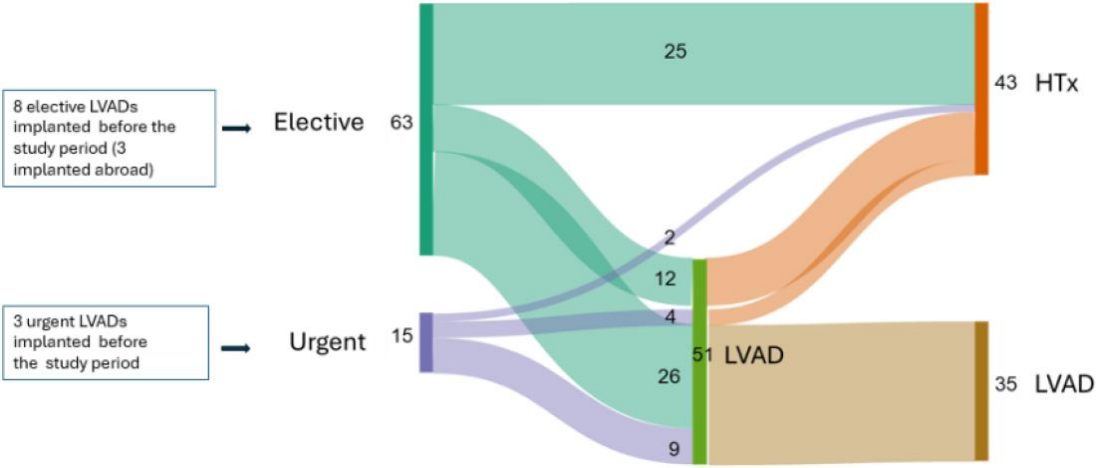
Patient flow diagram.

*Figure 1* illustrates the treatment pathways for 78 patients managed during the study period, categorized according to whether their first procedure at Helsinki University Hospital was elective or urgent. Eight patients who underwent HTx during the study period had received an LVAD before 2022 or in other hospital settings abroad, where cost data were unavailable. Four patients were transferred from Estonia, 3 of whom had LVAD before, and one of whom had LVAD in March 2022. As noted above, ‘Urgent’ is defined as being on ECMO before the index procedure.

Baseline characteristics for each group are summarised in *Table 1*. Age, sex, race, and body mass index (BMI) were comparable across groups. Group 1 included a higher proportion of patients in lower New York Heart Association classification (NYHA) classes, reflecting greater clinical stability—partly due to prior LVAD implantation before the study period. This group also included urgent HTx. In contrast, all patients in Group 3 were classified in higher NYHA classes and demonstrated worse pathology markers, consistent with advanced HF and the need for urgent intervention.

**Table 1:** Summary of study patient characteristics at baseline

	Group 1, HTx	Group 2, elective LVAD	Group 3, urgent LVAD
Sample size	36	30	12
Age, years^a^ (*SD)*	52.6 *(11.82)*	51.6 *(15.09)*	56.25 *(9.39)*
Male	28	23	9
Caucasian race (%)	100%	100%	100%
BMI (kg/m^2^) (*SD)*	25.85 *(3.34)*	24.23 *(4.18)*	25.86 *(3.66)*
Ischemic cause of heart failure	5	6	3
NYHA			
1	8	—	—
2	10	—	—
3	13	15	—
4	5	15	12
Serum sodium (mmol/l), *[P -Natrium]* (*SD)*	138.05 *(3.88)*	138.14 *(4.99)*	141.92 *(7.27)*
Serum creatinine (umol/l), *[P -Kreatiniini]* (*SD)*	130.81 *(57.09)*	117.13 *(32.04)*	102.23 *(62.75)*
Albumin (g/l) *[P -Albumiini]* (*SD)*	40.85 *(4.37)*	35.48 *(6.72)*	27.11 *(4.48)*
Total bilirubin (umol/l) *[P -Bilirubiini]* (*SD)*	19.97 *(12.31)*	20.78 *(12.92)*	18.23 *(4.48)*
Alanine aminotransferase *[U*/l *P -Alaniiniaminotransferaasi]* (*SD)*	34.54 *(15.56)*	94 *(95.18)*	147.62 *(181.81)*
6-minute walking test (m)	N = 20	N = 20	Not applicable
Meters (*SD)*	456.35 *(77.07)*	413.29 *(77.07)*	
% (*SD)*	79.25 *(17.07)*	68.95 *(16.09)*	

Abbreviations: BMI, body mass index; HTx, heart transplant; LVAD, Left Ventricular Assist Device; New York Heart Association classification system

^a^At the time of first operation during study period

### Survival

**Figure 2:**
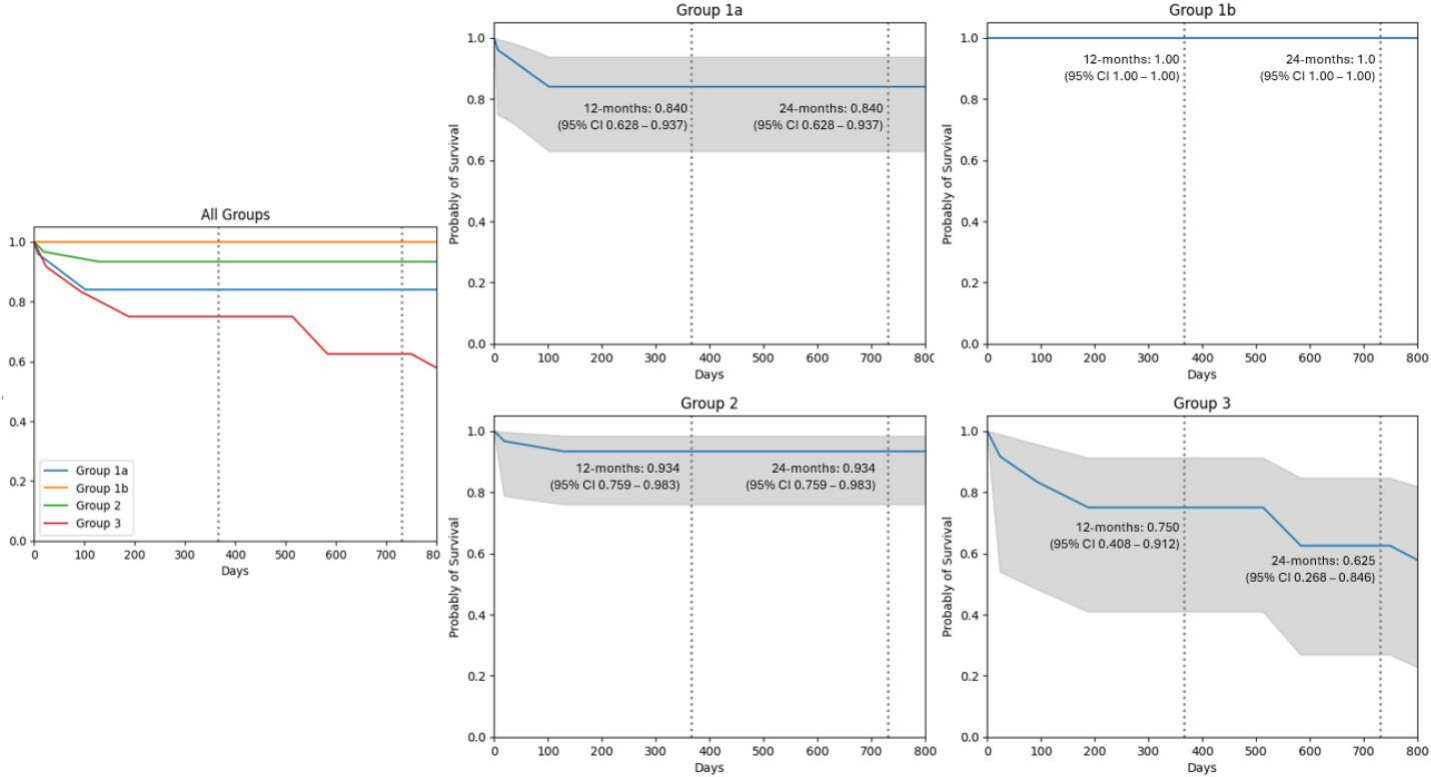
Survival on Kaplan–Meier. Abbreviations: KM, Kaplan–Meier; LVAD, left ventricular assist device. Grey indicates 95% confidence interval.

Survival outcomes, illustrated in *Figure 2*, found Group 1a (HTx without prior LVAD) had a 24-month survival rate of 84.0% (95% CI: 0.628–0.937), with most deaths (3 of 4) occurring within the first 3 months. Group 1b (HTx with prior LVAD) showed 100% survival throughout follow-up (95% CI: 1.000–1.000). Group 2 (elective LVAD) had two early deaths within 90 days, with survival stabilizing at 93.4% (95% CI: 0.759–0.983). Group 3 (urgent LVAD) had the poorest outcome, with progressive decline to 62.5% at 24 months (95% CI: 0.268–0.846). Ninety-five percent confidence intervals were computed for Groups 1a, 2, and 3, and are displayed in grey. As the confidence intervals between these groups overlap, the differences in 2-year survival were not statistically significant, likely due to the small sample size.

### Six-minute walking test

Both groups 1 and 2 showed steady improvement in 6MWT over the first year. In group 1, the distance walked increased from 456 meters (+/− 77 SD) and 79% (+/− 17.07%) predicted at baseline (*n* = 20) to 502 meters (+/− 53 SD) and 82% (+/− 8%) at 3 months (*n* = 7), reaching 593 meters (+/− 61 SD) and 99% (±15%) at 12 months (*n* = 5). Similarly, in group 2, the 6MWT improved from 413 meters (+/− 105 SD) and 69% (+/−16.09%) predicted at baseline (*n* = 20) to 471 meters (+/− 85 SD) and 76% at 3 months (*n* = 13), reaching 545 meters (+/− 110 SD) and 88% (+/− 12%) at 12 months (*n* = 20). The 6MWT is not typically performed on patients who require urgent care. In group 3, the distance walked at 3 months (*n* = 2) was 299 (+/−150) and 54.5% (+/−22%), which increased to 503.75 (+/−134) and 88.5% (+/−23%) by 12 months (*n* = 3).

### Cost analysis

**Figure 3:**
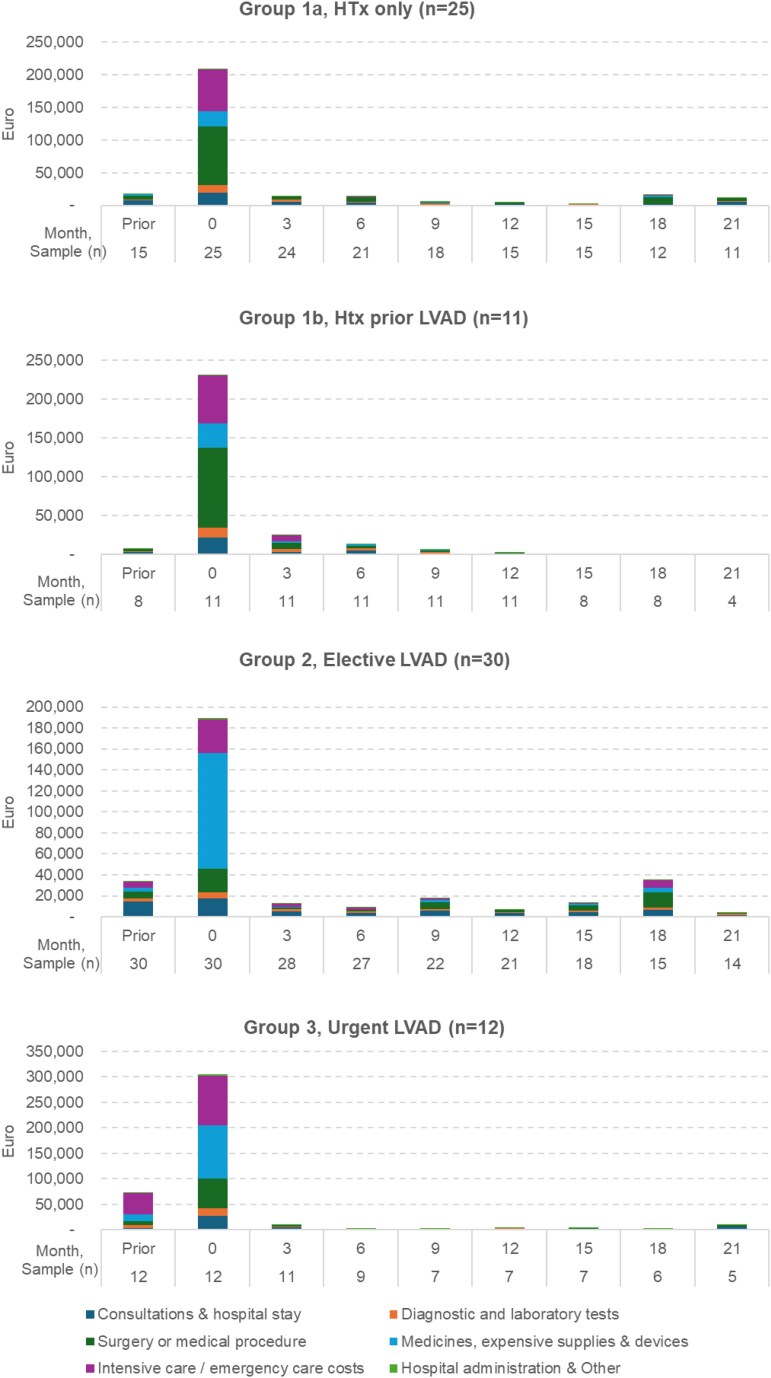
Mean costs captured between −3 and 24 months, with each time-period reflecting a 3-month period. Abbreviations: LVAD, left ventricular assist device. *Month describes the start of each 3-month time period. Sample size represents patients alive and with a follow-up cost reported within each time-period

The mean costs, by time-period and cost category is illustrated in *Figure 3* and show that all groups demonstrated a similar cost trajectory: a sharp cost peak where most costs are incurred at the time of intervention (0–3 months), followed by a marked decline. In the HTx groups (1a and 1b), costs were dominated by surgery and intensive care. Group 2 incurred the substantial medicine and equipment supply costs, attributed to the LVAD device and associated consumables. High costs at baseline in group 3 were driven by a combination of costly intensive care and device-related expenses. Post-intervention costs declined substantially across all groups after 3 months.

When individual patient cost curves were inspected, notable cost spikes were observed in the LVAD groups, where individual patients incurred substantially higher costs in a single period, related to subsequent heart transplants, and the cost returned to baseline after. In group 2 these occurred in months 9, 15, and 18. Similarly, in Group 3, an individual patient had a single substantial cost in month 21. Group 1a displayed one spike, where a single patient incurred substantial care costs between 3 and 9 months, and at month 18. In contrast, Group1b displayed minimal post-baseline cost variation, with no significant late spikes.

To further elucidate the reasons for cost differences in elective (group 2) vs urgent (group 3) LVAD patients, we looked at their postoperative in-hospital data. The ICU stays were shorter (median 5 vs 20.5 days), ventilator times were shorter (median 2 vs 13 days), and hospital stays were shorter (median 21 vs 55.5 days) (*P* < .001). Dialysis was less frequently needed (6.7 vs 50%, *P* < .01) and RVAD was less frequently used (3.3% vs 30%, *P* < .05) in elective vs urgent cases.

*Table 2* presents median and mean costs at 3, 6, and 12, and 24 months, excluding the prior costs. At all timelines-points the median costs are similar between both HTx groups (group 1a/1b) and elective LVAD (group 2) and substantially higher in the urgent LVAD group. At 3 months, the median costs per patient were €177 380 (IQR €121 900), €207 826 (IQR €83 398), €187 558 (IQR €67 664), and €293 355 (€158 721) in groups 1a, 1b, 2, and 3, respectively. Statistical analysis revealed significant variation in costs between groups, with Group 3 incurring significantly higher costs than Group 1a (*P* = .004) and Group 2 (*P* = .003). However, the difference between Group 3 and Group 1b was not statistically significant (*P* = .335), likely due to the smaller sample size in Group 1b. This pattern remained consistent at the 6-month follow-up. When this was repeated, pooling groups 1a and 1b, the difference between groups 1 and 3 was statistically significant. By 12 months, median costs rose to €186 561 (1a), €231 638 (1b), €209 660 (2), and €303 407 (3), though differences were no longer statistically significant due to greater cost variability and reduced sample sizes. At 24-months the median costs were €175 692 (1a), €219 843 (1b), €235 884 (2), and €314 548 (3), and no difference was detected between groups, and these conclusions did not change when groups 1a and 1b were pooled. The results at later time-points were limited by diminishing sample size over time, where patients were excluded for not reaching the follow-up period.

**Table 2:** Median and mean costs per patient

	Group 1a	Group 1b	Group 2	Group 3	All
3-month cost per patient					
Sample	25	11	30	12	
Median	€177 380	€207 826	€187 558	€293 355	*P* = .003
IQR	€121 900	€83 398	€67 664	€158 721	
*P*, relative to group 3	*P* = .004	*P* = .335	*P* = .003		
Mean	€210 110	€231 469	€189 459	€304 242	
SD	€99 817	€97 203	€35 513	€88 178	
6-month cost per patient					
Sample	24	11	28	11	
Median	€190 229	€221 266	€199 867	€316 139	*P* = .003
IQR	€144 675	€142 851	€62 318	€157 051	
*P*, relative to group 3	*P* = .004	*P* = .335	*P* = .003		
Mean	€226 142	€256 717	€198 851	€305 378	
SD	€105 663	€109 218	€39 793	€86 634	
12-month cost per patient					
Sample	18	11	27	7	
Median	€186 561	€231 638	€209 660	€303 407	*P* = .091
IQR	€117 523	€150 176	€65 559	€205 015	
Mean	€228 448	€276 409	€223 682	€295 751	
SD	€109 495	€122 679	€84 103	€91 581	
24-month cost per patient					
Sample	11	4	15	5	
Median	€175 692	€231 782	€235 884	€314 548	*P* = .526
IQR	€294 785	€81 288	€123 582	€202 897	
Mean	€262 798	€253 692	€264 027	€312 600	
SD	€147 075	€98 056	€118 282	€108 627	

Abbreviations: IQR, inter-quartile range; SD, standard deviation

The mean costs show greater variation at 12 and 24 months, skewed by individual patients who incurred substantial one-time costs in the follow-up period.

In a sensitivity analysis, the 3-month prior costs were included, and when the statistical test was repeated, group 2 was significantly more expensive than all the other groups at 3 and 6 months, but not at 12 or 24 months.

## Discussion

This study is the first to systematically report real-world, medium-term survival outcomes and cost data, indirectly comparing alternative treatment strategies for advanced HF in Finland. Two-year survival was high, above 80% in groups 1 and 2. In contrast, patients receiving LVAD as an urgent intervention had lower survival (62.5% CI 0.268–0.846). While the 95% confidence intervals overlap, the observed difference may be clinically meaningful and suggest that patient stability at the time of implantation could influence prognosis. Retrospective review of 6MWT data showed improved exercise capacity in all groups; however, the improvement was less pronounced in patients receiving LVAD as an urgent intervention compared to the other groups. Statistical tests were not performed due to the small sample size at follow-up.

Median costs were similar between groups 1 and 2, with most costs incurred in the first 3 months after surgery. While elective LVAD had higher equipment costs, it had lower surgical and intensive care costs compared to HTx, and median costs were similar between these three groups at all time points. Urgent LVAD was an outlier with substantially higher costs due to both expensive equipment and high-intensive care costs. Additionally, these patients incurred substantially higher costs three months before LVAD. Median costs were significantly higher in group 3 compared to group 1 (pooled) and group 2 at 3 and 6 months. Although median costs remained substantially higher at 12 and 24 months, the differences were not statistically significant, which may be due to greater cost variation at later time-points or reduced sample size. Patients requiring urgent LVAD were expected to have poorer outcomes and higher healthcare costs compared to those undergoing elective procedures. While HTx following LVAD was more expensive than primary HTx, reflecting added surgical complexity, it showed lower post-acute costs, with less cost variation over time. The other groups showed more skewed cost distributions, largely driven by a subset of patients who incurred one-time, substantial costs, mainly subsequent surgical procedures for HTx or a prolonged stay in intensive care. These findings underscore the heterogeneity in patients with advanced HF.

Survival outcomes are consistent with or better than those reported in the MOMENTUM 3 trial, which reported 2-year survival rates of 81.2%^20^ and 5-year survival rates of 58.4% with HM3.^13^ Higher 2-year survival in the elective LVAD group in this real-world study may be attributed to differentiating between elective and urgent LVAD, where earlier intervention is associated with better outcomes.

The costs reported here are also aligned with prior publications. A cost-effectiveness model,^16^ comparing costs and outcomes with LVAD to medical management, projected 5-year costs of £141 598 for HM3 LVAD recipients in the United Kingdom (UK). The higher costs observed in our real-world study likely reflect differences in healthcare system structures and methodological approaches. This analysis included all hospital-incurred costs, capturing expenses not directly linked to LVAD or HTx procedures. In contrast, economic models focus specifically on intervention-related costs.

This analysis is subject to several limitations. Patient heterogeneity makes direct comparison between treatment groups challenging, and small and decreasing sample size over time limits the statistical power of the analysis, reducing the ability to detect significant differences between treatment groups, even when clinically meaningful trends are observed. The follow-up time is relatively short, yet most expenses occurred within the first 3 months. Additionally, the complex health profiles and comorbidities typical of advanced HF patients limit the ability to attribute outcomes such as mortality or prolonged intensive care use to the initial procedure. Furthermore, classification of patients who transitioned between HTx and LVAD during the study period may introduce ambiguity in group assignment and outcome interpretation. As noted above, the costs reported reflect the direct cost captured at Helsinki University Hospital, which excludes costs incurred by primary care or other secondary care providers. Yet, all patients are on close follow-up and care in Helsinki University Hospital for the first post-procedural year after HTx and LVAD, and the vast majority even after that. As most hospitalizations, excluding some initial acute care takes place in Helsinki University Hospital, we believe that this analysis captures the high costs. Although prospective data collection would probably have yielded more complete follow-up of, e.g., 6-minute walk tests, the main parameters reported (costs and survival) could be reliably collected retrospectively.

Despite these limitations, this is the first study to report real-world cost and survival data over a 2-year period across key treatment pathways in advanced HF, and this analysis may offer valuable insights to inform decision-making, particularly when weighing the option of waiting for a donor heart versus proceeding with elective LVAD implantation, either as BTT or DT. These findings support the use of elective LVAD therapy in stable patients. Within group 3, urgent LVAD, a minority (3 out of 12) suffered from acute myocardial infarction, therefore, these patients suffered rapid subsequent deterioration and would not have been candidates for elective LVAD before this event. Nonetheless, earlier intervention with elective LVAD may have been a more cost-effective option for the other patients within the urgent LVAD group, associated with lower costs and potentially better outcomes compared to elective LVAD. These insights provide a foundation for optimizing patient selection and care pathways, in this high-risk population.

## Declarations

### Data Availability

The data underlying this article will be shared on reasonable request to the corresponding author.
